# Metabolic Variability in Micro-Populations

**DOI:** 10.1371/journal.pone.0052105

**Published:** 2012-12-27

**Authors:** Yuval Elhanati, Naama Brenner

**Affiliations:** 1 Department of Physics, Technion, Haifa, Israel; 2 Department of Chemical Engineering, Technion, Haifa, Israel; 3 Network Biology Research Lab, Technion, Haifa, Israel; University of Cambridge, United Kingdom

## Abstract

Biological cells in a population are variable in practically every property. Much is known about how variability of single cells is reflected in the statistical properties of infinitely large populations; however, many biologically relevant situations entail finite times and intermediate-sized populations. The statistical properties of an ensemble of finite populations then come into focus, raising questions concerning inter-population variability and dependence on initial conditions. Recent technologies of microfluidic and microdroplet-based population growth realize these situations and make them immediately relevant for experiments and biotechnological application. We here study the statistical properties, arising from metabolic variability of single cells, in an ensemble of micro-populations grown to saturation in a finite environment such as a micro-droplet. We develop a discrete stochastic model for this growth process, describing the possible histories as a random walk in a phenotypic space with an absorbing boundary. Using a mapping to Polya’s Urn, a classic problem of probability theory, we find that distributions approach a limiting inoculum-dependent form after a large number of divisions. Thus, population size and structure are random variables whose mean, variance and in general their distribution can reflect initial conditions after many generations of growth. Implications of our results to experiments and to biotechnology are discussed.

## Introduction

Biological cell populations exhibit a broad distribution of phenotypes, even if genetically homogenous. Such variability has been observed in practically every single-cell phenotypic property that was measured, including cell size, protein content, division rate and more. Although this is an issue of general interest in biology, populations of microorganisms have provided important model systems for developing novel experimental single-cell techniques and applying theoretical analysis of the problem. Some of the major topics of recent work have been understanding the mechanisms underlying phenotypic variability [1]–[5], modeling the distribution of phenotypes in populations of cells [6]–[8], and revealing the role of phenotypic variability in the fate and survival of microbial populations [9]–[14].

In the standard approach to the problem, from single cell properties that have some stochastic components emerge the statistical properties of a large (practically infinite) population. Within this approach, variability in division rate seems to be a most important property for population dynamics, and its implications have been widely studied, especially in the context of fluctuating environments [15]–[19].

However, a population of growing and dividing cells is not a statistical ensemble of independent individuals. The environment always plays an important role in real population growth; even without direct interactions, as cells grow they continually modify their environment and thus indirectly affect other population members [20], [21]. These considerations become even more important in many biologically relevant situations where growth is physically or biochemically constrained by the environment and the limit of an infinite population is never reached. Inheritance and memory in the population can then cause a delicate dependence on initial conditions that can be more important over finite growth times than the asymptotic dynamics.

When the environment is taken into account, another single-cell property becomes important in addition to the division rate - the yield with respect to growth resources. For example, in growth under flow in a well-mixed environment, such as that described by an ideal chemostat, although the fastest-growing cells take over, the yield determines the population density at equilibrium [22]. Spatial structure in the environment can result in coexistence of high-yield and high-growth-rate phenotypes [23], whereas models for density-dependent selection show that high carrying capacity, which is analogous to high yield, can be selected under some conditions [24], [25]. The picture that emerges from these works is that the role of yield with respect to growth resource is strongly dependent on the details of the environment and how exactly it constrains the population.

Recent advances in microfluidic and microdroplet technology have enabled to grow “micro-populations”, intermediate-sized populations that grow over intermediate lengths of time [26]–[30]. These experimental systems enable to follow the temporal dynamics of the populations and to accumulate statistics of a large number of such populations with well controlled initial and environmental conditions. While obviously promising many important practical applications, these advances at the same time define a new regime for studying cell populations and highlight some questions of fundamental nature: What is the variability between different micro-populations? What is the dependence on initial conditions and how does it decay with time and system size? One is led to consider the statistical properties of an ensemble of finite populations, rather than those of single cells in an infinite population. For example, the dependence on initial conditions in constrained micro-chemostat growth was recently studied [31]. Using a model of growth-rate variability of single cells, it was found that sometimes the effect of initial conditions persists indefinitely, while in other circumstances it can take a very long time until this effect decays.

In this work we study theoretically the effects of variability in division rate and in yield on population dynamics in limited environments such as finite droplets. In many cases of biological relevance the yield is inversely correlated with division rate at the single cell level as both vary across the population [23], [32]–[34]. Experimental evidence supporting such a “metabolic tradeoff” was reviewed in [33] and in the Supplement of [34]. Recent theoretical work has shown that accounting for metabolic tradeoffs between these two cellular properties can give rise to nontrivial behavior at the population level, even in a deterministic model in a simple chemostat-like environment [34]. Previous work suggested that high-yield growth can be considered a cooperative strategy that models evolutionary dilemmas [23], [33], while here we are interested primarily in the statistical effects and in long-term traces of initial conditions on finite populations. We develop a stochastic model with emphasis on tracking histories of individual equally prepared micro-populations to characterize their distribution at the end of growth. While this work is of basic and theoretical nature, there are implications to experimental measurements and to practical biotechnology that arise and are discussed briefly at the end.

## Model

A single metabolic network defined by its enzymes, metabolites and biochemical reactions can support many different modes of metabolic flux at steady state growth [35], [36]. These modes are generally associated with different cellular properties such as division rate and yield with respect to growth resources. Considering the large variability observed in practically any physiological property that was measured at the single cell level in microorganisms, it is only natural to assume that the metabolic state, and with it the yield, are also variable properties in such a population. This assumption is supported by experiments on bacteria grown in continuous culture, showing a broad range of operational modes of the metabolic system [37]. In addition to their direct measurements, which is relatively difficult, the implications of yield variability can possibly be measured in terms of the population dynamics and statistics under constrained conditions as described below.

We consider a population of microorganisms with a range of metabolic states, each characterized by a different yield towards a growth-limiting resource. In the model the population will thus be represented as a mixture of several different metabolic states, each characterized by a division rate 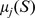 and a yield 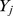. A single growth-limiting resource, a nutritional substrate 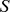, describes the environment. The state space of the entire system is characterized by the number of cells of each phenotype and the amount of resource at a given time 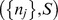. Cells of state 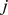 can divide with probability 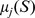 per unit time, in which case the resource is depleted by an amount 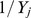. The different states supported by the metabolic network are generally of finite lifetime and therefore cells can change their metabolic state. These changes can be described by transitions from state 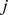 to 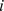 with probability 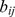 per unit time, in which case the substrate remains unchanged. The full model is completely specified by the following master equation:
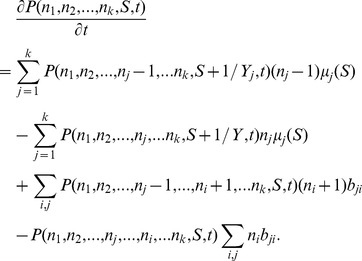



In the present analysis, however, we will be interested in timescales over which transitions can be neglected and they will therefore not be included in the model. A metabolic tradeoff is represented at a phenomenological level, by imposing some relation between the growth rate and yield at the single cell level.

## Results

### Deterministic (Average) Dynamics

**Figure 1:**
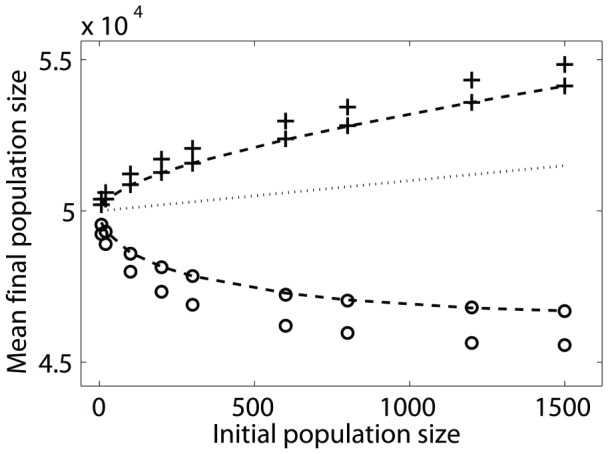
Average final vs. initial population size in micro-populations grown to saturation of resource. Dotted line: a population with a uniform yield. Symbols: Monte Carlo results for two-state populations with variability in yield and in growth rate. Dashed lines: analytic approximations relevant only for special parameter values. Crosses: Monte Carlo simulation for “metabolic tradeoff” 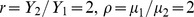 (lower crosses), 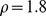 (upper crosses). circles: variable yield positively correlated with division rate 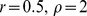 (upper circles), 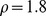 (lower circles).



A homogeneous population growing in a microdroplet, starting with initial conditions of cell number and resource 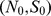, will grow to a stopping time 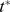 when the resource is depleted. The stopping time and the final number of cells 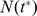 is determined by the number of divisions and the amount of resource taken up for each division:
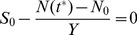
(1)where 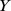 is the uniform yield. For typical droplets the final population size is on the order of 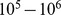 cells, and the starting initial number 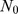, typically in the range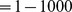, is relatively very small. The final population size is therefore dominated by 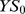, and its dependence on 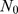 negligible (see Figure 1; dotted line). However, if the population is heterogeneous in its properties, generally its composition will change over time and with it the averaged yield. This will affect both the stopping time and the final number of cells. To see this, consider the deterministic approximation to the model dynamics: as long as there is still growth resource, these dynamics are described by the equations

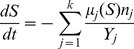
(2)

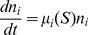
(3)where 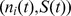 is the average trajectory of the system over time. The stopping condition, 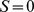, analogous to Eq. (1) can then be written, taking into account this variability:



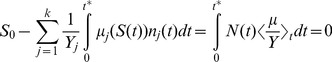
(4)Here 
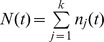
 is the total number of cells at time 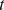, and we have defined the average of any property 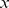 over the population at time 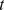 as 
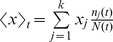
. The stopping time and the final cell number are therefore seen to depend on the average resource uptake rate 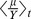 along the trajectory, which makes them generally history-dependent quantities. Note that if cells differ only by their division rate but have all the same yield, the relation (1) is recovered; although the population composition changes with time, the mean uptake rate remains the same.

It is instructive to consider a simplified population with only two phenotypes and with a step-function dependence of the division rate on the substrate: 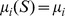 when 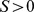 and 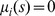 when 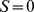. We assume initial conditions to be distributed around a symmetric mixed population so on average the initial amount of each phenotype will be half of the total initial population. Eq. (4) then reads.
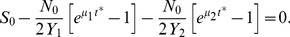
(5)


This equation can generally be solved numerically for the stopping time and from that the final number of cells can be found. For a special choice of division rates 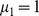, 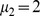 one may solve analytically the resulting quadratic equation in 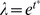. Defining the ratio between the yields of the two cell states as 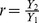 the quadratic equation reads.
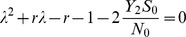
revealing a dependence on one additional dimensionless parameter, 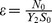, which represents the initial population size relative to the approximate number of divisions supported by the medium. From the physical solution of this equation, 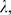 we find an explicit expression for the final number of cells as a function of the initial number:
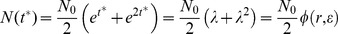
(6)where the function 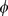 gives a generally nonlinear dependence on the initial number of cells 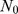. In many experimental cases of interest 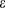 is very small; droplets are started with a small number of cells and the amount of resource allows a large number of cell divisions before saturation. Expanding in this small parameter we find the approximation



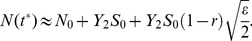
(7)Now imagine an experiment in which the initial droplet cell content 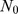 is varied while other parameters are held fixed. Then the correction to the final cell number induced by metabolic variability will depend nonlinearly on the initial number, to leading order as the square root of 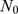, with coefficients that can result in an increasing or decreasing function depending on the ratio between yields. Recall that we have marked by 2 the faster-growing phenotype, and therefore the ratio between yields, 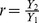, defines the correlation between division rate and yield in single cells. A ratio smaller than one, 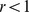, describes a metabolic tradeoff, namely cells that grow faster have a smaller yield. Such a negative correlation between growth and yield has been observed in several cases for microorganisms (reviewed in [33], [34]). Then, the final number of cells is an increasing function of the initial conditions (Figure 1, upper increasing dashed line). By contrast if the yield is positively correlated with the division rate, 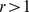, a decreasing curve is found (Figure 1, lower decreasing dashed line). These analytical results are relevant for a special choice of division rates which render the equation solvable, however we expect that the qualitative nature of the solutions to be insensitive to the exact values of division rates. For comparison Figure 1 (symbols) shows also the results of a Monte-Carlo simulations of the two-state population that grows in a microdroplet, giving the final cell number directly without any approximation. For a growth-rate ratio of 2, the analytic results are recovered (symbols overlapping with dashed lines); for a different non-integer ratio, the qualitative features remain. (+, metabolic tradeoff; o, yield and division rate positively correlated).

### Stochastic Analysis by Phenotype-space Trajectories

The results for the averaged dynamics show that yield variability can significantly modify the dependence of the final mean number of cells on the initial number. It is expected that the variability among populations will show an even more pronounced dependence; to investigate this variability we consider the stochastic dynamics of the model. Here the object of interest is the probability distribution of the system among its possible states, 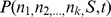 which obeys the Master Equation (see Eq. (1)), so that for a given initial condition the explicit time dependence of the distribution can be computed. However, to describe the ensemble of populations grown to saturation we need much less information and one can formulate the problem in a simpler way. The key observation is that in order to characterize the distribution of populations at the end of this finite-time growth one needs only the sequence of divisions that make up the trajectories, and not the time points at which they occurred. Therefore one may study the geometry of random trajectories in phenotype-space; we develop this approach for the toy two-state model discussed above, but the basic idea can be generalized to multiple states.

The growth of a micro-population with two cell states is pictured as a random walk on a discrete two-dimensional phenotype plane 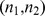 with the axes representing the number of cells of each type. Time units of this random walk will be taken as times between divisions (these time units are not constant but vary randomly). The initial condition is 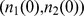, and at each point 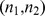 there is a probability 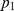 for the next division to be of type 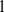, in which case the trajectory will advance one step along the 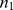 direction; and a probability 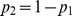 for it to be of the other type, in which case it will advance along the 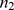 direction. To find 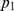 we approximate the divisions as Poisson processes with probabilities per unit time 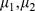 to divide. The probability that the next division is of type 1 and occurs after exactly time 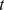 is given by.
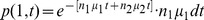



**Figure 2:**
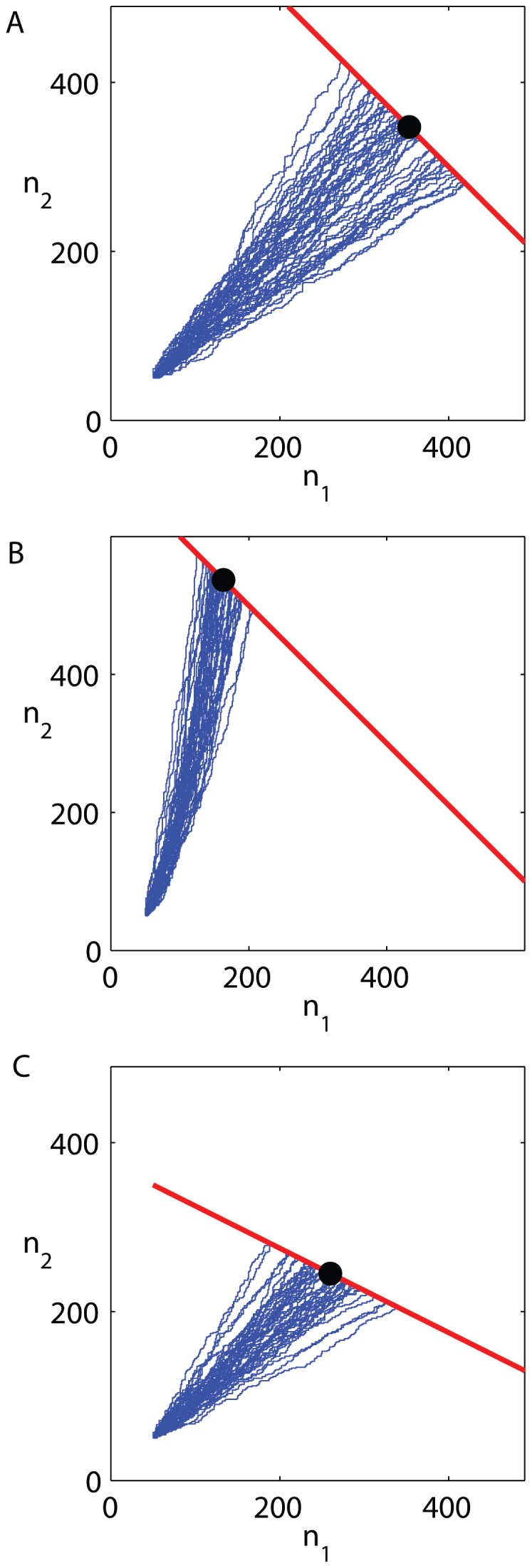
Trajectories in phenotypic space for heterogeneous micro-populations grown to saturation of resource. Each point in the plane 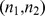 represents the number of cells of each type in the population. Division of type 1 increases 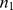 by one and corresponds to the trajectory advancing along the 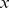-direction, and similarly for type 2. All three panels show trajectories that start from an initial population of 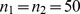. (A) symmetric types (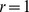, 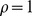). Trajectories are equally likely to proceed along 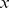 or 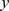, and the stopping condition is of slope (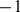). (B) Types differ by their division rates (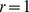, 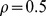), causing the trajectories to be biased towards the faster growing type. (C) Types differ in their yield with respect to the finite resource (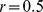, 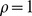), causing the stopping line to be of slope different from (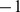); growth will stop after a variable total number of divisions depending on trajectory, since each type consumes a different amount of resource at division.

Therefore the probability for the next division to be of type 1, regardless of the time it occured, is just the integral over 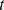 of the above expression,
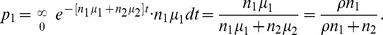



In contrast to a simple random walk, where the probability to move in each direction is the same everywhere, here it depends on the position in the plane, namely on the number of cells of each type. It also depends globally on the ratio 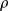 between their division rates. To describe in phenotypic plane the stopping condition at depletion of the resource, we note that the two cell types consume a quantity 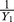 or 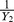 respectively of this resource in each division. Therefore the geometric place of all points 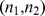 that correspond to the stopping condition is.
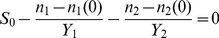
(8)which can be written as: 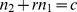, the “stopping line” (using the yield ratio 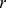 and defining the constant 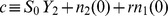). The form of the equation describing the stopping line shows that if 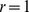 all trajectory endpoints have the same final number of cells 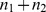, while for 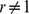 the final number of cells varies along the stopping line. Figure 2 illustrates the space and the dynamics by showing some individual trajectories of micro-population growth: starting from a given initial condition 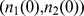, each trajectory shows the history of a single population which, depending on the order of divisions of each of the two types, proceeds randomly in the phenotypic plane until it hits the stopping line where all trajectories are absorbed. In Figure 2A the two cell types are uniform in their properties and the random walk is therefore symmetric in the two directions. The final number of cells is deterministic and only the final population composition - what fraction of each type - is variable, depending on the details of the random trajectory, with a symmetric distribution. Figure 2B shows that non-uniform division rates of the two types cause a bias in the trajectories and they become curves in the plane. Still, the final number of cells is deterministically fixed because both types have the same yield. In contrast Figure 2C shows that non-uniform yields modify the slope of the stopping line, and therefore each trajectory on that line has a different final number of cells. In this graphical representation, it is clear that the two metabolic properties - division rate and yield - play separate and different roles in the problem: the first determines the bias of trajectories while the second determines the slope of the stopping line. Any assumed relation between them can be inserted as additional input to the model.

#### Probability distributions at the stopping line

The probability distribution at the stopping line is determined by the probabilities of trajectories arriving at each point; this is what allows us to leave out of the discussion the temporal structure of trajectories. As we have seen, the composition of the final population, namely the number of 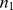 cells, is always a random variable even if the cell types are identical in their phenotypic properties. Our goal is to calculate 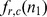, the probability density function of 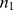 on the stopping line 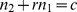, given an initial condition 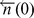, or at least to estimate its moments.

We first consider the simpler case where the yield in the population is uniform (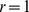 or 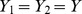) and the division rate is uniform (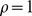 or 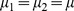), corresponding to Figure 2A. The total number of cell divisions in all trajectories is fixed at 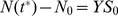, and we have two types of events - division of type 1 or 2 - occuring with the same probability out of a total given number of events. This can be mapped to a classic problem in probability theory known as Polya’s Urn [38], [39]. In Polya’s original problem, white and black balls are drawn at random from an urn; each time one ball is chosen it is returned back to the urn with an additional ball of the same color. In our analogy the probability that out of 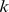 draws from the urn 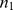 will be white is equivalent to the probability that out of 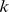 divisions 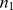 will be of type 1. This probability is the well known Beta-Binomial distribution:
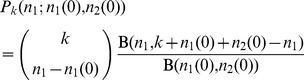
(9)where 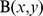 is the Beta function. Using the moments of this distribution, the ratio between its standard-deviation and mean is



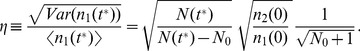
(10)This ratio, representing the relative width of the final distribution, does not decay with an increasing number of divisions; in the limit 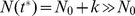 it converges to a quantity proportional to the inverse square root of the inoculum size. An additional factor represents the initial composition, namely how many cells of each type were in the inoculum. Assuming that in a typical experiment this initial composition is averaged over some set of possible preparations, there remains still a strong dependence on the inoculum size: 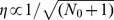. At the extreme, starting many copies of an initial population with a single cell of each type, the final ensemble of populations has all possible values of 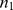 between 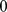 and 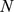 with equal probability (a uniform distribution), no matter how many generations have passed. In contrast to our intuition from many repetitions of a coin-flipping, there is no convergence after a long time to a distribution that is strongly peaked around 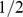. This result illustrates an important property of all models studied here: for a large number of cell divisions, the distribution tends to a limit whose shape is independent of the number of divisions but still maintains a fingerprint of the initial population size [40].

A generalization of Polya’s urn can be constructed for the more general case of variable division rates, (illustrated in Figure 2B). Since the ratio 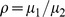 is the only relevant parameter for the phenotype-plane trajectories, we may approximate it by the ratio of two large integers 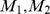 and map the problem to an urn in which each cell of type 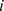 is represented by 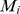 balls of color i. If each ball chosen at random is returned to the urn with an additional 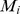 balls of the same color, the ratio of probabilities for choosing each color remains faithful to the ratio of division rates, and the number of cells of each color in the urn at any given time is proportional to the number of cells of the corresponding type. Formulation of this construction can be found in Text S1.

**Figure 3:**
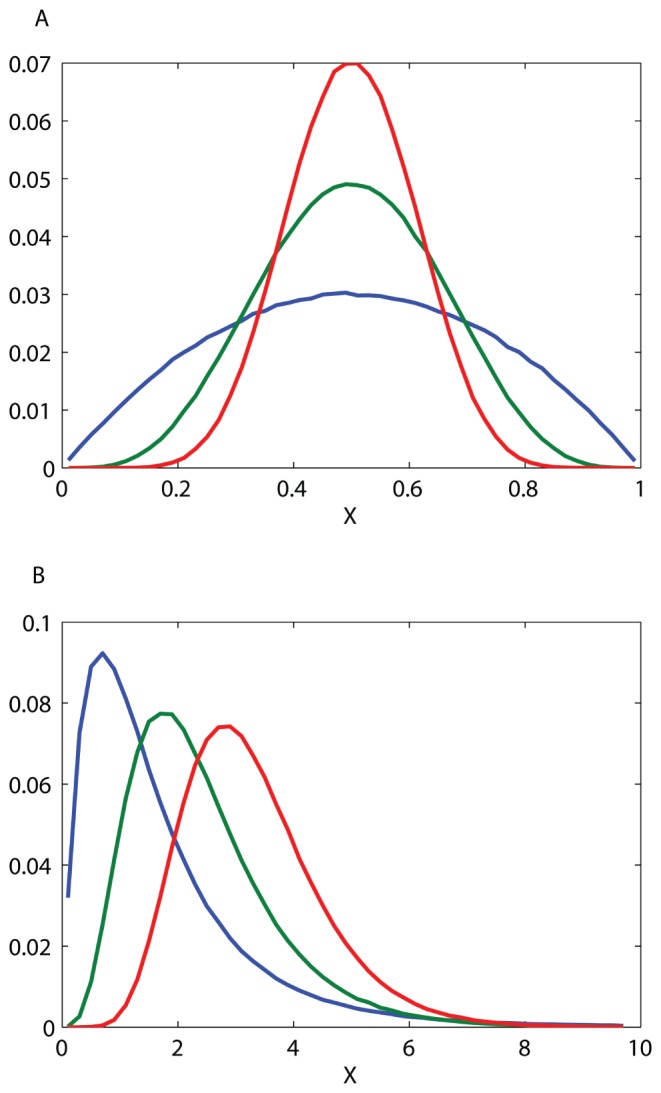
Scaled distribution of the number of cells of metabolic type 1 in the final population. All distributions are for symmetric initial composition, equal yields and a large number of divisions. Different distributions in a plot are for different initial populations 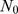 (Blue - 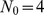, Green - 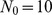, Red - 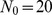). These distributions are plotted as a function of the scaling variable 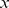 (see text for details), and their shape does not depend on the number of divisions but does depend on the initial number of cells. (A) The two types have the same growth rate and are therefore equal in all their properties. Population composition varies only because individual trajectories are composed of different sequences of divisions of the two types. Because of the symmetry between types, all distributions are symmetric around 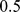. (B) The two types have different growth rates, and the distribution of final composition becomes skewed.

The limiting distribution shape is revealed in term of scaling variables. Figure 3 shows several such limiting distributions for the case of uniform yield as a function the scaling variable 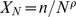. For uniform division rate (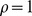) this variable reduces to 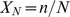, the fraction of type-1 divisions, and the limit distribution is just the bounded Beta distribution 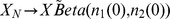. For non-uniform division rate the scaling variable is a more abstract mathematical quantity, and its distribution converges as 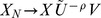 where 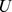 and 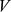 are two Gamma distributed random variables - 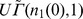, 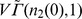. This distribution has no closed form density but has known moments (see Text S1 for details). The relative width of these distributions in both cases, equal and variable division rates, decreases as the inoculum size increases.

In the example above, Eq. (10) and the distributions of Figure 3 manifestly show how the size of the initial preparation affects in the variation in population composition when yield is uniform. This is somewhat analogous to the effect of population bottlenecks on variability through genetic drift. Next we consider the more interesting case of a population with variable yield, in which not only the composition but the total final number of cells is itself a random variable.

#### Yield variability and distribution of final population size

In terms of the graphical phenotypic plane representation, we are interested in trajectories that end on a line with slope 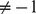, since r is now different from one (see Eq. (8)). In our model trajectories in phase plane can advance only in one of two directions: increasing 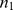 or 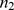 (this property is lost if transitions between phenotypes are accounted for). Using the stopping condition and this monotone property of the trajectories, one can find a relation between the cumulative probability function of 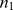 with non-uniform yields 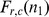 and the uniform case discussed in the previous section, but with an effective value of 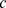:
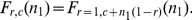
(11)


**Figure 4:**
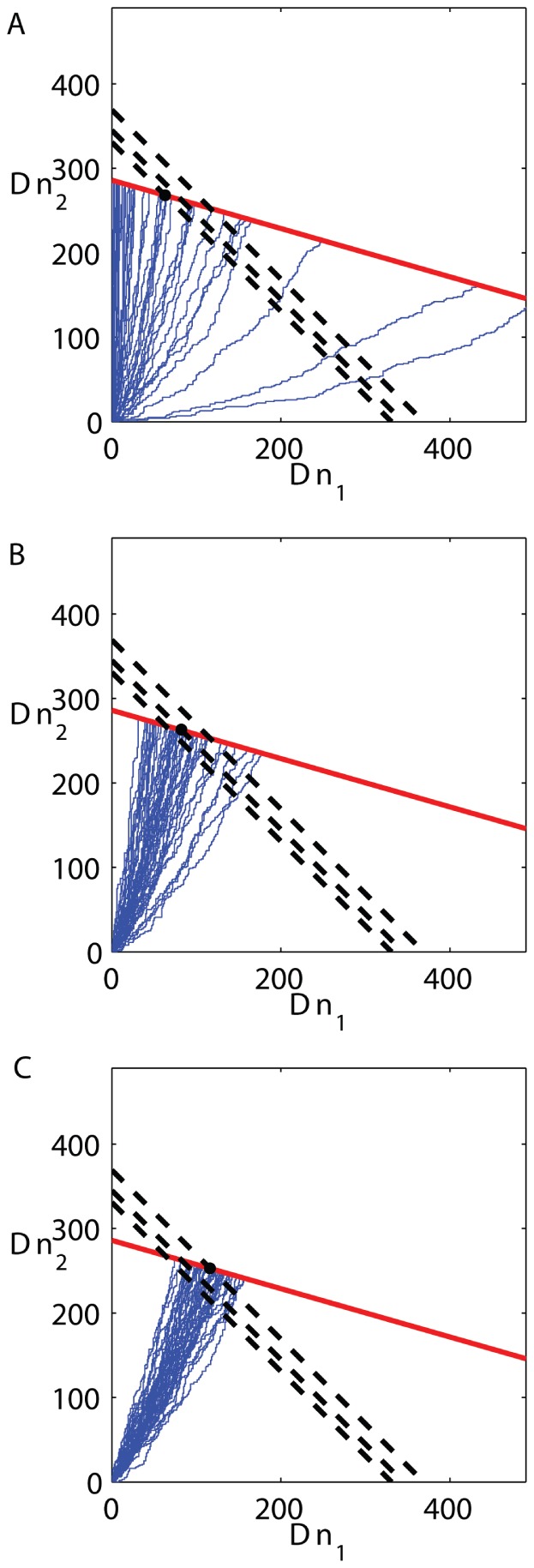
Relating trajectories of uniform and variable yield populations. All gray trajectories, ending on the solid red line (variable-yield stopping line) at points corresponding to 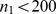, build up the cumulative probability for the final population to have less than 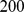 cells of type 1. Due to the monotone property of trajectories, they all cross also the dashed line (uniform-yield stopping line) that passes through the point 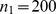 obeying the same ocnstraint and thus the cumulative probability is the same. The parameters of the two lines are simply related through 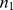 (see Eq. (11)).

This relation provides the key to computing the distributions in the variable-yield population from the uniform-yield results presented above. It is illustrated graphically in Figure 4.

When yields are variable, each point on the stopping line corresponds to a different final population size. It is determined by the 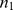 coordinate of that point and by the initial conditions:
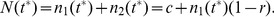
(12)


This relation is used to define the scaling variable 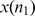, which reveals the limiting distribution shape for a large number of divisions (see Text S1 for details). The mean and variance of this distribution are found by inverting this scaling variable as 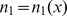, as follows:
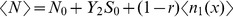
(13)

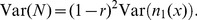
(14)


These expressions show that the mean final population size obtains an additional term from yield variability (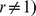 which can be negative or positive depending on the correlation between division rate and yield (see also Figure 1). The variance in final population size, of course, becomes positive from yield variability with a variance that increases with the extent of this variability, represented by 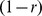 in this two-state model. A detailed derivation of these results is presented in Text S1.

**Figure 5:**
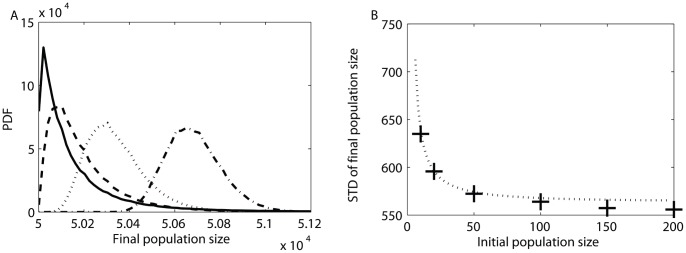
Trajectories in phenotypic space illustrating dependence on inocculum size. Results of Monte Carlo simulations are shown for initial population sizes of 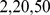 equally divided between the two types. Axes are number of divisions. Parameters: 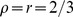. All trajectories end on the stopping line (red line) which has an asymmetric slope; trajectories ending at different points on this line have a different final population size (dashed lines). The mean of all trajectory endpoints on the line is marked with a black circle. It can be seen that as the inoculum size increases, going from (A) through (B) to (C), the spread of trajectories decreases and their mean crosses a dashed line corresponding to a higher number of cells.

The dependence on the inoculum size is incorporated in the moments of the random variable 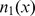. To illustrate this dependence graphically in phenotype space, we show in Figure 5 sets of trajectories starting at different inoculum sizes with all other parameters held fixed. In this figure, for ease of comparison, the axes are the number of divisions during growth 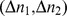 rather than the absolute number of cells of each type. The difference in growth rates causes a bias of trajectories towards 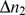, the faster growing type. For small inoculum sizes (Figure 5A), an initial step in the 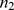 direction can rapidly amplify itself causing a heavy bias of the mean towards this type; because this rapidly growing type is less efficient, the mean number of cells at the stopping line is smaller, namely the stopping line intersects a dashed line 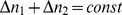 with a smaller value of the constant. At the same time, this amplified sensitivity causes a wide spread of the trajectories. As inoculum size increases, the relative effect of this bias at the stopping line becomes smaller (Figures 5B, 5C); the trajectories are less spread-out and the mean final number of cells increases.

In several special cases of interest the moments of the distribution of final population size can be explicitly calculated or approximated, for example the case of equal growth rates, or growth rate ratio of exactly 2. Analytic calculation is harder in the general case of variable growth rates and variable yields. Details of the calculations for special cases which allow solution are presented in the Text S1. In any case the moments can be calculated directly from averages over the limiting distribution by numerically inverting the relation defining the scaling variable.

**Figure 6:**
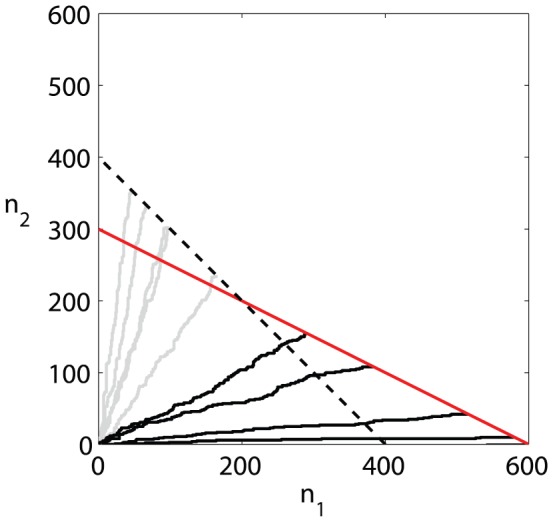
Final population size for a heterogeneous micro-population with metabolic tradeoff. (A) Distributions of the final populations size from simulations with division rate ratio 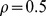 and yield ratio 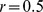, for different initial population sizes - 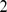 cells (solid line), 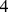 cells (dashed line), 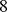 cells (dotted line), 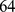 cells (dash-dot line). In (B) we can see the Standard deviation of the final population size as function of initial population size, in good agreement with the analytic approximation.

In Figure 6 we show several examples of the distribution of final population size (A), and approximations for the standard deviation which are in good agreement with the simulation (B) and decreases as a power law of the inoculum size.

The stochastic analysis of the model allows the computation of variance and higher moments, analytically in some cases and numerically in others. The average is consistent with the deterministic approximation solution presented earlier, however it is expressed by moments of another random variable which is not generally available in closed form. In the deterministic analysis it was possible to develop a controlled approximation for the limit of large number of divisions, which is not easily done in the general stochastic equations.

## Discussion

Recent advances in microdroplet and microfluidic technology for growing cell populations have the potential to provide basis for many practical applications. At the same time, they open up an interesting regime for cell population behavior that has previously been unexplored. The intermediate sizes of populations, the control over initial conditions and the availability of an ensemble of populations equally prepared, offer to think about these micro-populations as repeated experiments of mesoscopic sized systems and to consider the stochastic properties of this ensemble of finite populations.

Cell populations have a structured and dynamic phenotypic composition in terms of their metabolic properties, and in particular their growth rate and their yield with respect to growth-limiting resources. This structure can strongly influence the dynamics of the population in the intermediate-size regime. We have formulated and solved a theoretical model that relates metabolic variability of cells to measurable statistical properties illustrating the emergence of novel behavior in this regime of micro-populations. In the absence of asymptotic takeover that results from infinite-time competition, the presence of both efficient and fast-growing phenotypes creates a dependence between the dynamics of the population composition and the effective consumption rate of the resource, resulting in a variability of the final population composition and size. Moreover, even the mean final population size exhibits an inoculum dependency which does not disappear after a large number of divisions. We have demonstrated this effect for a two state model with large ratios of yields and divisions rates. With smaller, more realistic ratios of the metabolic parameters, the two state model produce a smaller but qualitatively identical effects. However, modeling a more realistic situation would also require including a broader range of phenotypes. These more realistic models remain subject for future work.

As a theoretic approach to analyzing this regime of population growth we have constructed a mapping to a probabilistic problem, a generalization of Polya’s Urns [41], which allowed us to compute stochastic properties of the population. In this class of problems it has been shown that limiting distributions appear which are independent of the number of trials but do depend on the initial conditions; in the case of a dividing population, it means that statistical properties of the ensemble of populations converge to a limit after a large number of divisions, which still reflects the initial condition of the inoculum.

All of these predictions obtained in our work can be tested in experiments on microdroplets. For example, the dependence of the final distribution on initial condition (number of cells in preparation) but its independence on number of divisions (amount of resource in each droplet) can be tested directly. At the present state of the technology both these parameters can be controlled to good accuracy. The dependence of variance on inoculum size can be easily measured [27]. The extent of yield variability, represented in our model by 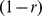, is expected to be sensitive to the type of growth medium and other details of growth conditions [42]. A systematic investigation of these statistical properties as a function of growth parameters can indirectly provide much information about actual yield variability in a microorganism population and its dependence on environmental parameters.

The limiting environment and the finite growth time in microdroplets bring into light the importance of yield variability, which has been largely overlooked in previous studies. In unconstrained populations the fastest dividing cells take over and thus division rate is often equated with the Darwinian concept of “fitness”. In micro-population, on the other hand, the yield becomes a selective parameter – the yield composition of the population changes by the time the resource is depleted, with the higher-yield cells having a larger representation in the population regardless of their growth rate. This effect can be used to select high-yield cells via cycles of growth to saturation followed by mixing (B. Teusink, unpublished results, 2012). Such a controlled growth protocol can have implications to biotechnology by providing an empirical way to maximize biomass yield in microorganisms building on existing yield variability in the population. Developing such protocols is subject for future theoretical and experimental research.

## Supporting Information

Text S1 (PDF)
