# Supplementary material for: Metabolic Variability in Micro-Populations
Source: PLoS One. 2012 Dec 27;7(12):e52105. doi: 10.1371/journal.pone.0052105 (PMC3531411; doi:10.1371/journal.pone.0052105)
Supplement: Text S1 — (PDF) [file pone.0052105.s001.pdf]

Supporting Information

Mapping dividing populations to Urn Models Consider Polya's original problem, with an urn containing initially w_0 white balls and b_0 black balls. At each draw a ball is chosen randomly and uniformly, and returned with another identical ball back to the urn [1]. The number of white ball draws, W_k, out of k total draws was shown by Polya and Eggenberger [2] to follow the Beta-Binomial distribution:

$$P_k(W_k = w; w_0, b_0) = \binom{k}{w} \frac{\mathrm{B}(w + w_0, k - w + b_0)}{\mathrm{B}(w_0, b_0)} \tag{1}$$

Where the Beta function is $\mathrm{B}(x, y) = \int_0^1 t^{x-1}(1-t)^{y-1}dt$. As explained in the main text, this maps to our problem when the growth rates are identical and the above quantity is exactly the probability of the population reaching $n_1 = w_0 + w$ cells of type 1 after $N = k$ total divisions, for an initial state of $(n_1(0), n_2(0)) = (w_0, b_0)$.

The moments of the distribution Beta-Binomial distribution are

$$\langle W \rangle = k \frac{w_0}{w_0 + b_0}$$

$$\mathrm{Var}(W) = k \frac{w_0 b_0 \left(w_0 + b_0 + k\right)}{\left(w_0 + b_0\right)^2 \left(w_0 + b_0 + 1\right)}$$

Leading the the main text result on the ratio between standard-deviation and mean.

For large values of N this distribution approaches the continues Beta distribution. Formally $W_N \to NW$, where $W \sim \mathrm{Beta}(n_1(0), n_2(0))$. This can also be written as $W = \frac{V}{V+U}$, with U, V random variables with Gamma distributions: $U \sim \Gamma(n_1(0), 1)$, $V \sim \Gamma(n_2(0), 1)$.

Generalizing to a non-symmetric model, the growth rates can be considered as weights that factor the probability of the next division. In the language of Polya urns this can be represented by modifying the number of balls that corresponds to a cell. Denoting the number of draws of each kind by (W_N, B_N), the initial state is taken as $(w_0, b_0) = (\mu_1 n_1(0), \mu_2 n_2(0))$. Following a draw of a ball, we return it with μ_1 or μ_2 new balls according to its color. Effectively, now we draw balls from an urn that has a distribution of white and black balls that is biased according to the asymmetry of growth rates, therefore the probability of picking a white ball will be proportional to the probability of type 1 cell performing the next division. Consequently, n_1 - the number of cells with growth rate μ_1, will have the same distribution as $\frac{w_0 + W_N}{\mu_1}$. In this case

the limit distribution is unbounded: $W_N \to N^{-\rho} W$, where $W = \mu_1 U^{-\rho} V$ and $U \sim \Gamma(w_0/\mu_1, 1) = \Gamma(n_1(0), 1)$, $V \sim \Gamma(b_0/\mu_2, 1) = \Gamma(n_2(0), 1)$. [3]

The limiting distributions are more easily written in terms of the number of divisions $\widetilde{n}_i = n_i - n_i(0)$ instead of the absolute number of cells. Since the two differ by a constant, the only difference will be in the mean of the distribution. Summarizing both cases the number of divisions of type 1 has a known limit distribution $X_N = N^{-\rho}\widetilde{n}_1 \longrightarrow X$ as appearing in the main text. Formally: we denote the cumulative distribution of $\widetilde{n}_1$ by $F_{1,N}$, where the subscript 1 represents the ratio between the yields of the two types, one in the symmetric case under consideration. Then this function is related to the cumulative distribution of X_N and through it approximated by the function of the random variable X:

$$F_{1,N}(\widetilde{n}_1; n_1(0), n_2(0)) = F_{X_N}\left(\frac{\widetilde{n}_1}{N^\rho}; n_1(0), n_2(0)\right) \to F_X\left(\frac{\widetilde{n}_1}{N^\rho}; n_1(0), n_2(0)\right) \tag{2}$$

With the limit taken formally as $\frac{N}{\max(n_1(0), n_2(0))} \to \infty$.

Final size distributions with yield variability

Here we calculate in the distribution of $\widetilde{n}_1$, number of divisions of type 1, on the stopping line $\widetilde{n}_2 + r\widetilde{n}_1 = \widetilde{c} (= S_0 Y_2)$, relating its cumulative distribution function $F_{r,\widetilde{c}}$ with the the one for the symmetric case we calculated earlier $F_{1,N}$. Since the trajectories are monotone, all trajectories that stop on the stopping line with less then $\widetilde{n}_1$ divisions of type 1, also stop on the symmetric line that passes through this point, with less then $\widetilde{n}_1$ division of this type (see illustration in the Appendix of the main text). Therefore, the cumulative distribution for this point can be taken from the corresponding cumulative distribution of the symmetric problem, using $\widetilde{N} = \widetilde{n}_1 + \widetilde{n}_2 = \widetilde{n}_1 + \widetilde{c} - r\widetilde{n}_1 = \widetilde{c} + \widetilde{n}_1(1-r)$ for the total number of divisions :

$$\begin{aligned} F_{r,c}(\widetilde{n}_1; n_1(0), n_2(0)) &= F_{1,\widetilde{c}+\widetilde{n}_1(1-r)}(\widetilde{n}_1; n_1(0), n_2(0)) \\ &= F_{X_{\widetilde{N}=\widetilde{c}+\widetilde{n}_1(1-r)}}\left(\frac{\widetilde{n}_1}{(c+\widetilde{n}_1(1-r))^\rho}; n_1(0), n_2(0)\right) \\ &\approx F_X\left(\frac{\widetilde{n}_1}{(\widetilde{c}+\widetilde{n}_1(1-r))^\rho}; n_1(0), n_2(0)\right) \end{aligned} \tag{3}$$

Where the last approximation is valid when $\widetilde{c} \gg \max(n_1(0), n_2(0))$, corresponding to a small initial condition relative to the growth potential of the medium (see definition of ε in the deterministic model). In this limit we can

find the connection between the probability density functions:

$$\begin{aligned}
f_{r,\widetilde{c}}(\widetilde{n}_1;n_1(0),n_2(0))dn_1 &= \frac{d}{d\widetilde{n}_1}F_{r,\widetilde{c}}(\widetilde{n}_1;n_1(0),n_2(0))d\widetilde{n}_1 \\
&= \frac{d}{d\widetilde{n}_1}F_{1,\widetilde{c}+n_1(1-r)}(n_1;n_1(0),n_2(0))d\widetilde{n}_1 \\
&\approx \frac{d}{d\widetilde{n}_1}F_X\left(\frac{\widetilde{n}_1}{(\widetilde{c}+\widetilde{n}_1(1-r))^\rho};n_1(0),n_2(0)\right)d\widetilde{n}_1 \\
&= f_X\left(\frac{\widetilde{n}_1}{(\widetilde{c}+\widetilde{n}_1(1-r))^\rho};n_1(0),n_2(0)\right)\frac{d}{d\widetilde{n}_1}\left(\frac{\widetilde{n}_1}{(\widetilde{c}+\widetilde{n}_1(1-r))^\rho}\right)d\widetilde{n}_1 \\
&= f_X\left(x;n_1(0),n_2(0)\right)dx \qquad (4)
\end{aligned}$$

With the change of variables:

$$x(\widetilde{n}_1) = \frac{\widetilde{n}_1}{(\widetilde{c}+\widetilde{n}_1(1-r))^\rho} \qquad (5)$$

Thus relating the moments:

$$\begin{aligned}
\langle \widetilde{n}_1^k \rangle_{r,\widetilde{c}} &= \int \widetilde{n}_1^k f_{r,\widetilde{c}}(\widetilde{n}_1;n_1(0),n_2(0))dn_1 \\
&= \int \widetilde{n}_1^k(x) f_X\left(x;n_1(0),n_2(0)\right)dx \\
&= \langle \widetilde{n}_1^k(x) \rangle_X \qquad (6)
\end{aligned}$$

When $\widetilde{n}_1(x)$ is the inverse function to (5).

If now we return to the absolute number of cells, instead of number of divisions, all the central moments, including the variance, are the same and the mean shifts by the initial number of cells. Overall, the mean and variance of the total population are:

$$\langle N\rangle_{r,\widetilde{c}} = N_0+\left\langle \widetilde{N}\right\rangle_{r,\widetilde{c}} = N_0+\widetilde{c}+(1-r)\langle \widetilde{n}_1\rangle_{r,\widetilde{c}} = N_0+S_0Y_2+(1-r)\langle \widetilde{n}_1(x)\rangle_X \qquad (7)$$

$$\mathrm{Var}(N)_{r,\widetilde{c}} = \mathrm{Var}(\widetilde{N})_{r,\widetilde{c}} = (1-r)^2\mathrm{Var}(\widetilde{n}_1)_{r,\widetilde{c}} = (1-r)^2\mathrm{Var}(\widetilde{n}_1(x))_X \qquad (8)$$

Final population size distribution for the two-state model with equal growth rates In the case of equal growth rates ($\rho = 1$) X follows the Beta distribution, which has a compact support and a PDF:

$$f_X(x;n_1(0),n_2(0))dx = \frac{x^{(n_1(0)-1)}(1-x)^{(n_2(0)-1)}}{\mathrm{B}(n_1(0),n_2(0))}dx \tag{9}$$

And the scaling variable in (5) is $x = \frac{\widetilde{n}_1}{\widetilde{c}+\widetilde{n}_1(1-r)}$. Inverting the scaling relation is straightforward in this case $\widetilde{n}_1 = \frac{\widetilde{cx}}{1-x(1-r)}$, and so the moments of $\widetilde{n}_1$ can be calculated using the moments of X. Starting with the average of $\widetilde{n}_1$:

$$\langle \widetilde{n}_1 \rangle_{r,c} = \left\langle \frac{\widetilde{c}x}{1-x(1-r)} \right\rangle_X = \left\langle \widetilde{c}\sum_{k=1}^{\infty}(1-r)^{k-1}x^k \right\rangle_X = \widetilde{c}\sum_{k=1}^{\infty}(1-r)^{k-1}\left\langle x^k \right\rangle_X$$

We can now use the known moments of the Beta distribution:

$$\left\langle x^k \right\rangle_X = \frac{\mathrm{B}(n_1(0)+k,n_2(0))}{\mathrm{B}(n_1(0),n_2(0))}$$

And so obtain the expansion:

$$\langle \widetilde{n}_1 \rangle_{r,c} = \widetilde{c}\sum_{k=1}^{\infty}(1-r)^{k-1}\frac{\mathrm{B}(n_1(0)+k,n_2(0))}{\mathrm{B}(n_1(0),n_2(0))} \tag{10}$$

Which is the expansion of the ordinary hypergeometric function:

$$\langle \widetilde{n}_1 \rangle_{r,c} = \widetilde{c}\frac{n_1(0)}{n_1(0)+n_2(0)}\,{}_2F_1(1,n_1(0)+1,n_1(0)+n_2(0)+1;1-r) \tag{11}$$

When r is close enough to 1, we can truncate the expansion:

$$\begin{aligned}\langle \widetilde{n}_1 \rangle_{r,c} &\approx \widetilde{c}\frac{n_1(0)}{n_1(0)+n_2(0)}\left[1+\frac{n_1(0)+1}{n_1(0)+n_2(0)+1}(1-r)\right] \\ &= \widetilde{c}q\left[1+\frac{qN_0+1}{N_0+1}(1-r)\right] \end{aligned} \tag{12}$$

Where in the last line we used $n_1(0) = qN_0$.

Using $\widetilde{N} = c + \widetilde{n}_1(1-r)$, this result gives immediately the dependence of the mean number of total divisions on the inocculum size and composition:

$$\langle N \rangle = N_0 + S_0Y_2 + (1-r)S_0Y_2q\left[1+\frac{qN_0+1}{N_0+1}(1-r)\right]$$

Now turning to the variance of the final population, we write a similar expansion for the second moment:

$$\langle \widetilde{n}_1^2 \rangle_{r,c} = \left\langle \left[\frac{cx}{1-x(1-r)}\right]^2 \right\rangle_X = \widetilde{c}^2\sum_{k=2}^{\infty}(k-1)(1-r)^{k-2}\left\langle x^k \right\rangle \tag{13}$$

$$\begin{aligned}\left\langle \widetilde{n}_1^2 \right\rangle_{r,c} &= \widetilde{c}^2 \sum_{k=2}^{\infty} (k-1)(1-r)^{k-2} \frac{\mathrm{B}(n_1(0)+k, n_2(0))}{\mathrm{B}(n_1(0), n_2(0))} \\ &= \widetilde{c}^2 \frac{n_1(0)}{n_1(0)+n_2(0)} {}_2F_1'(1, n_1(0)+1, n_1(0)+n_2(0)+1; 1-r) \quad (14)\end{aligned}$$

Which can also be truncated:

$$\left\langle \widetilde{n}_1^2 \right\rangle_{r,c} \approx \widetilde{c}^2 \left[\left\langle x^2 \right\rangle_X + 2 \left\langle x^3 \right\rangle_X (1-r) \right] \quad (15)$$

Therefore the variance of the number of cells is

$$\begin{aligned}\mathrm{Var}(\widetilde{n}_1)_{r,c} &= \left\langle \widetilde{n}_1^2 \right\rangle_{r,c} - \left\langle \widetilde{n}_1 \right\rangle_{r,c}^2 \\ &\approx \widetilde{c}^2 \left[\left\langle x^2 \right\rangle_X + 2 \left\langle x^3 \right\rangle_X (1-r) \right] - \widetilde{c}^2 \left[\left\langle x \right\rangle_X^2 + 2 \left\langle x \right\rangle_X \left\langle x^2 \right\rangle_X (1-r) \right] \\ &= \widetilde{c}^2 \frac{q(1-q)}{N_0+1} \left[1 + 4 \frac{qN_0+1}{N_0+2} (1-r) \right] \quad (16)\end{aligned}$$

Leading to the variance of N:

$$\mathrm{Var}(N) = (1-r)^2 \, \mathrm{Var}(\widetilde{n}_1)_{r,c} = (S_0 Y_2)^2 \, \frac{q(1-q)}{N_0+1} (1-r)^2 \left[1 + 4 \frac{qN_0+1}{N_0+2} (1-r) \right]$$

For small enough r:

$$\eta \equiv \frac{\sqrt{\mathrm{Var}(N)}}{\langle N \rangle} = \frac{S_0 Y_2 \sqrt{\frac{q(1-q)}{N_0+1}} \, (1-r)}{S_0 Y_2} = (1-r) \sqrt{\frac{q(1-q)}{N_0+1}}$$

Meaning the standard-deviation over the mean decreases as $\eta \propto 1/\sqrt{(N_0+1)}$. This is in agreement with the earlier calculation using the discrete case Beta-Binomial distribution

Final population size distribution for the two-state model with variable special values of growth rates The model with variable growth rate can be solved for the special case of $\rho = 0.5$. As argued previously, we expect that the qualitative nature of the solution will not depend strongly on the exact values of the growth rates; therefore we solve this case as an illustratio. The scaling relation (5) is:

$$x = \frac{\widetilde{n}_1}{(\widetilde{c} + (1-r)\widetilde{n}_1)^{\frac{1}{2}}} \tag{17}$$

Which can be inverted as

$$\widetilde{n}_1^2 - (1-r)x^2\widetilde{n}_1 - x^2\widetilde{c} = 0$$

$$\widetilde{n}_1 = \frac{(1-r)x^2 + \sqrt{(1-r)^2x^4 + 4x^2\widetilde{c}}}{2} = \frac{1-r}{2}x^2 + \sqrt{\widetilde{c}}x\sqrt{1 + \frac{(1-r)^2x^2}{4\widetilde{c}}} \tag{18}$$

Using (18) we can again calculate the mean and variance using the first few moments of X [3]:

$$\langle x^k \rangle = \frac{\Gamma(n_1(0) - \rho k)\Gamma(m_s(0) + k)}{\Gamma(n_1(0))\Gamma(n_2(0))} \tag{19}$$

The first moment of the number of divisions of type 1 is then found

$$\langle \widetilde{n}_1 \rangle = \left\langle \frac{1-r}{2}x^2 + \frac{x}{2}\sqrt{(1-r)^2x^2 + 4\widetilde{c}} \right\rangle_X = \frac{1-r}{2}\langle x^2 \rangle_X + \sqrt{\widetilde{c}}\,\langle x g(x) \rangle_X \tag{20}$$

Where we have defined $g(x) = \sqrt{1 + \frac{(1-r)^2x^2}{4c}}$. Using the fact that c is very large enables to write it as a combination of the moments of X:

$$\langle \widetilde{n}_1 \rangle \approx \sqrt{\widetilde{c}}\,\langle x \rangle_X + \frac{1-r}{2}\langle x^2 \rangle_X \tag{21}$$

Leading to the result for the total number N:

$$\langle N \rangle = N_0 + S_0Y_2 + (1-r)\sqrt{S_0Y_2}\frac{\Gamma(n_1(0) - 0.5)\Gamma(n_2(0) + 1)}{\Gamma(n_1(0))\Gamma(n_2(0))} + \frac{(1-r)^2}{2}\frac{\Gamma(n_1(0) - 1)\Gamma(n_2(0) + 2)}{\Gamma(n_1(0))\Gamma(n_2(0))}$$

This approximation appears as a series in the extent of yield variability $(1-r)$. A direct expansion in $\varepsilon = \frac{N_0}{Y_2S_0}$, as we had in the deterministic case, cannot be formulated as a controlled approximation here since the effect of the initial size enters through the moments of X. Next we turn to the second moment:

$$\begin{aligned} \langle \widetilde{n}_1^2 \rangle &= \left\langle \left(\frac{1-r}{2}x^2 + \frac{x}{2}\sqrt{(1-r)^2x^2 + 4\widetilde{c}} \right)^2 \right\rangle_X \\ &= \left\langle \left(\frac{(1-r)^2}{4}x^4 + \frac{x^2}{4}\left((1-r)^2x^2 + 4\widetilde{c}\right) + \frac{1-r}{2}x^3\sqrt{(1-r)^2x^2 + 4\widetilde{c}} \right)^2 \right\rangle_X \end{aligned}$$

$$= \frac{(1-r)^2}{2}\left\langle x^4\right\rangle_X + \widetilde{c}\left\langle x^2\right\rangle_X + \frac{1-r}{2}\left\langle x^3\sqrt{(1-r)^2x^2+4\widetilde{c}}\right\rangle_X \quad (22)$$

Again approximating for large values of initial substrate:

$$\left\langle \widetilde{n}_1^2\right\rangle \approx \widetilde{c}\left\langle x^2\right\rangle_X + (1-r)\sqrt{\widetilde{c}}\left\langle x^3\right\rangle_X + \frac{(1-r)^2}{2}\left\langle x^4\right\rangle_X \quad (23)$$

We can obtain a result for the variance.

$$\begin{aligned}
\mathrm{Var}(\widetilde{n}_1)_{r,c} &= \left\langle \widetilde{n}_1^2\right\rangle_{r,c} - \left\langle \widetilde{n}_1\right\rangle_{r,c}^2 \\
&= \frac{(1-r)^2}{2}\left\langle x^4\right\rangle_X + \widetilde{c}\left\langle x^2\right\rangle_X + (1-r)\sqrt{\widetilde{c}}\left\langle x^3 g(x)\right\rangle_X \\
&= (1-r)\sqrt{\widetilde{c}}\left(\left\langle x^3 g(x)\right\rangle_X - \left\langle x^2\right\rangle_X \left\langle xg(x)\right\rangle_X\right) + \widetilde{c}\left(\left\langle x^2\right\rangle_X - \left\langle xg(x)\right\rangle_X^2\right)
\end{aligned}$$

Putting these results together we obtain for the variance for the total final population:

$$\begin{aligned}
\mathrm{Var}\left(N\right)_{r,c} &\approx S_0Y_2(1-r)^2\left(\left\langle x^2\right\rangle_X - \left\langle x\right\rangle_X^2\right) + (1-r)^3\sqrt{S_0Y_2}\left(\left\langle x^3\right\rangle_X - \left\langle x^2\right\rangle_X\left\langle x\right\rangle_X\right) \\
&\quad + \frac{(1-r)^4}{2}\left(\left\langle x^4\right\rangle_X - \frac{1}{2}\left\langle x^2\right\rangle_X^2\right) \\
&\approx S_0Y_2(1-r)^2\left(\frac{\Gamma(n_1(0)-1)\Gamma(n_2(0)+2)}{\Gamma(n_1(0))\Gamma(n_2(0))} - \left(\frac{\Gamma(n_1(0)-0.5)\Gamma(n_2(0)+1)}{\Gamma(n_1(0))\Gamma(n_2(0))}\right)^2\right) \\
&= S_0Y_2(1-r)^2\left(\frac{n_1(0)(n_2(0)+1)}{(n_1(0)-1)} - \left(\frac{n_2(0)\Gamma(n_1(0)-0.5)}{\Gamma(n_1(0))}\right)^2\right) \quad (24)
\end{aligned}$$

References

[1] W Feller. *An introduction to probability theory and its applications.* Wiley series in probability and mathematical statistics: Probability and mathematical statistics. Wiley, 1971.

[2] F Eggenberger and G Pólya. Über die Statistik verketteter Vorgänge. *Zeitschrift für Angewandte Mathematik und Mechanik*, 3(4):279–289, 1923.

[3] Svante Janson. Limit theorems for triangular urn schemes. *Probability Theory and Related Fields*, 134(3):417–452, May 2005.
